# Tracking Object-State Representations During Real-Time Language Comprehension by Native and Non-native Speakers of English

**DOI:** 10.3389/fpsyg.2022.819243

**Published:** 2022-03-04

**Authors:** Xin Kang, Haoyan Ge

**Affiliations:** ^1^Research Centre for Language, Cognition and Language Application, Chongqing University, Chongqing, China; ^2^School of Foreign Languages and Cultures, Chongqing University, Chongqing, China; ^3^School of Education and Languages, Hong Kong Metropolitan University, Hong Kong, Hong Kong SAR, China

**Keywords:** object-state, mental representation, visual world paradigm, non-native speakers, native speakers

## Abstract

The present “visual world” eye-tracking study examined the time-course of how native and non-native speakers keep track of implied object-state representations during real-time language processing. Fifty-two native speakers of English and 46 non-native speakers with advanced English proficiency joined this study. They heard short stories describing a target object (e.g., *an onion*) either having undergone a substantial change-of-state (e.g., *chop the onion*) or a minimal change-of-state (e.g., *weigh the onion*) while their eye movements toward competing object-states (e.g., a chopped onion vs. an intact onion) and two unrelated distractors were tracked. We found that both groups successfully directed their visual attention toward the end-state of the target object that was implied in the linguistic context. However, neither group showed anticipatory eye movements toward the implied object-state when hearing the critical verb (e.g., “*weigh/chop*”). Only native English speakers but not non-native speakers showed a bias in visual attention during the determiner (“*the*”) before the noun (e.g., “*onion*”). Our results suggested that although native and non-native speakers of English largely overlapped in their time-courses of keeping track of object-state representations during real-time language comprehension, non-native speakers showed a short delay in updating the implied object-state representations.

## Introduction

There is extensive evidence that native speakers anticipate what comes next in language comprehension (Altmann and Mirković, 2009; Kuperberg and Jaeger, 2015). For example, Altmann and Kamide (1999) found that visual attention was directed to the target object before it was explicitly mentioned in the language. However, as most of the available studies focused on native speakers, it remains debated whether non-native speakers anticipate upcoming information in language comprehension to the same extent as native speakers.

Existing studies on non-native speakers have primarily focused on the use of morphosyntactic features and grammatical knowledge during language comprehension, such as gender (Lew-Williams and Fernald, 2010; Dussias et al., 2013; Hopp, 2013; Bañón and Martin, 2021), syntactic or semantic ambiguity (Frenck-Mestre and Pynte, 1997; Wilson and Garnsey, 2009; Dussias et al., 2010), and phonological forms (DeLong et al., 2005; Martin et al., 2013). Several studies have revealed that non-native speakers were not as quick or as accurate as native speakers in making predictions (Kaan et al., 2010; Lew-Williams and Fernald, 2010; Grüter et al., 2012; Martin et al., 2013; Kaan, 2014). But other studies observed native-like predictive processing in non-native speakers (Dahan et al., 2000; Dussias et al., 2013; Hopp, 2013; Foucart et al., 2014; Trenkic et al., 2014). The differences between native and non-native language comprehension are often attributed to factors such as complexity of linguistic subdomains (Clahsen and Felser, 2006) and variability in non-native speakers’ proficiency of and exposure to the target language (Dussias et al., 2013; Kaan, 2014; Hopp and Lemmerth, 2016; Li et al., 2020).

Nonetheless, these studies have not considered the recruitment of non-linguistic information in language comprehension. According to mental/situation models (e.g., Johnson-Laird, 1983; van Dijk and Kintsch, 1983) and perceptual symbol systems (Barsalou, 1999, 2008), language comprehension involves not only the activation of linguistic knowledge but also situations and mental representations grounded in sensorimotor experiences (but see Mahon and Caramazza, 2008). For example, Zwaan and colleagues showed that language comprehenders were faster to verify pictures that matched the implied orientation (Stanfield and Zwaan, 2001), shape (Zwaan et al., 2002), visibility (Yaxley and Zwaan, 2007) than pictures that mismatched (see also Taylor and Zwaan, 2009; Horchak et al., 2014). Marino et al. (2014) revealed that viewing photos and reading nouns of natural graspable objects modulated motor responses. In addition, previous studies demonstrated that toddlers with low reading skills and limited use of language activate mental representations of objects in language comprehension, suggesting that the recruitment of non-linguistic information might not be dependent on the proficiency of language (e.g., Engelen et al., 2011; Johnson and Huettig, 2011; Bobb et al., 2016).

However, compared with the number of studies on the role of non-linguistic information in native language processing, there were fewer studies on the role of non-linguistic information in the case of a non-native language (Kühne and Gianelli, 2019). Some studies support the idea that during the processing of non-native language, non-linguistic information is activated (Kogan et al., 2020). For example, Dudschig et al. (2014) revealed that bilinguals activated motor responses when they processed action and emotion words in their non-native language. Buccino et al. (2017) showed that fluent speakers of a second language processed graspable nouns in a second language like in their native language. Parker Jones et al. (2012) revealed that bilinguals and monolinguals differed in brain activation during picture naming and reading aloud. De Grauwe et al. (2014) found that non-native speakers activated motor and somatosensory brain areas when they were presented motor verbs in the non-native language like native speakers. Nonetheless, there is limited evidence on the timing of activating non-linguistic information in language comprehension by native and non-native speakers.

In the present study, we examined the activation of mental representations of objects in real-time language processing by native and non-native speakers of English. Specifically, we investigated to what extent native and non-native speakers of English overlapped in their time courses of keeping track of object-states as language unfolded. According to the “intersecting object histories” (IOH) hypothesis, dynamic changes in objects across time are used as primitives of event representations (Altmann and Ekves, 2019). Multiple representations of objects are activated and updated during language processing (e.g., Hindy et al., 2012, 2015; Solomon et al., 2015; Kang et al., 2019, 2020; Horchak and Garrido, 2020; Hupp et al., 2020; Lee and Kaiser, 2021; Misersky et al., 2021; Santin et al., 2021). Kang et al. (2020) revealed that native speakers of English shifted their eye movements between two competing object-state representations of the target object in real-time language comprehension. In their study, participants were asked to listen to short stories in 2 × 2 conditions, such as “*The chef will* ***chop/weigh*** *the onion. **But first/And then**, he will smell the onion*” while viewing a visual stimulus showing two competing states of the target object (e.g., a chopped onion vs. an intact onion) and two distractors. They found that participants preferred to look at the changed object-state (e.g., a chopped onion) when it matched the implied end-state of the target object compared to when it mismatched the implied end-state in the first sentence (e.g., ***chop*** *vs.* ***weigh*** *the onion*). Interestingly, the bias of visual attention occurred at the end of the second sentence when the target object was explicitly mentioned.

In the present study, we tested two competing hypotheses. One hypothesis is that non-native speakers should be as quick and as accurate in activating and updating object-state representations as native speakers in real-time language processing since the construction of event representation is not subject to how good one is at understanding or using the language (e.g., Bobb et al., 2016). An alternative hypothesis is that non-native speakers and native speakers show differences in keeping track of object-state representations supported by cross-linguistic differences in event categorization and perception (e.g., Papafragou et al., 2006; Brown and Gullberg, 2010; Papafragou and Selimis, 2010; Flecken et al., 2015; Aveledo and Athanasopoulos, 2016).

We opted to test these hypotheses by using the visual world paradigm that has been used in previous studies on real-time language processing (Tanenhaus et al., 1995). In this paradigm, participants are instructed to view or manipulate objects in the “visual world” (either in real-world or on a computer screen) while their eye movements toward these objects are recorded as they listen to short stories that describe events related to these objects. We expect that if native and non-native speakers keep track of event representations to the same extent during real-time language processing, they should have the same time courses of directing their visual attention toward the implied object-state as the language unfolds.

## Method

### Participants

A 52 native speakers and 46 non-native speakers of English participated in this study. None of them reported impairment in vision or hearing. Non-native speakers of English were native speakers of Cantonese and were studying at a research university in Hong Kong where English was used as the instruction language. All participants signed written informed consent before joining this study and received cash compensation for their participation. Table 1 presents the demographics of participants. The sample size was determined based on a previous study (Kang et al., 2020). Compared to the previous study, the present study has fewer conditions (2 vs. 4) but more trials per condition than the previous study (12 vs. 9). We performed a power simulation using *simr* package (Green and MacLeod, 2016). Simulation results showed that with 45 participants and 24 trials the statistical power for Degree of Change was 80%.

**TABLE 1:** Demographics and language background of native and non-native speakers of English.

Variables	Native English speakers	Non-native English speakers
Gender (M/F)	1.11	1.05
Age (mean)	18–42 (21)	18–27 (20)
Age starting English (mean)	NA	0–10 (3)
Years of learning English (mean)	NA	10–24 (17)
Mean IELTS score (SD)	NA	7.78 (0.34)

### Materials

We constructed 24 pairs of linguistic stimuli that described either a minimal or a substantial change-of-state event. Each stimulus contained four sentences. The first three sentences set up the context of the story. The fourth sentence was the critical sentence that described either a substantial change-of-state or a minimal change-of-state (“*The rabbit is weighing/chopping* ***the onion)***, followed by a negative clause (e.g., “*not smelling* ***the onion***”). For example:

**(A) Minimal Change-of-State Event (ME):** *The rabbit has a bowl, a bottle of pills, and* ***an onion***. *She was going to smell* ***the onion***. *Then she changed her mind. The rabbit is weighing* ***the onion***, *not smelling* ***the onion***.
**(B) Substantial Change-of-State Event (SE):** *The rabbit has a bowl, a bottle of pills, and* ***an onion***. *She was going to smell* ***the onion***. *Then she changed her mind. The rabbit is chopping* ***the onion***, *not smelling* ***the onion***.

**FIGURE 1:**
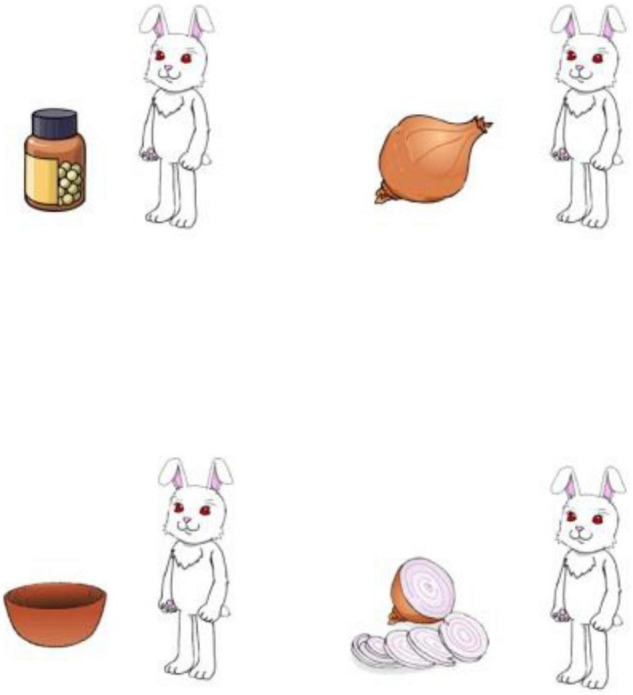
Example visual stimulus. Participants heard sentences such as “*The rabbit has a bowl, a bottle of pills and an onion. She was going to smell the onion. Then she changed her mind. The rabbit is weighing/chopping the onion, not smelling the onion”.*

The linguistic stimuli were recorded in a soundproof booth by a male native speaker of British English at 44.1k Hz sampling rates with 16 bits resolution. Each stimulus was scaled to 70 dB SPL in mean intensity using Praat (Version 6.0.39; Boersma and Weenink, 2018). Each pair of linguistic stimuli was associated with a visual stimulus that depicted the protagonist with four objects using clipart images (Figure 1). The locations of the objects were counter-balanced across visual stimuli.

### Procedure

Two counter-balanced lists were created for the experiment. Each list consisted of 24 experimental trials and 20 filler trials. Half of the experimental trials described a minimal change of the object-state (e.g., “*weighing the onion*”) [**ME condition, as in (A)**] and the other half a substantial change of the object-state (e.g., “*chopping the onion*”) [**SE condition, as in (B)**]. Filler trials followed the same structure as the experimental trials. The trials were presented in a pseudorandomized order. In each trial, participants viewed the visual display and heard the auditory stimuli simultaneously. Eye movements on the visual stimulus were tracked during the experiment. The total time of the experiment was about 20 min.

Tobii TX300 was used to collect eye movement data. The sampling rate was 300 Hz from both eyes. Freedom of movement was 37 × 17 cm at a 65 cm distance and gaze accuracy was 0.47 degrees. Tobii Studio was used to display the stimuli and collect the data. Experimental trials in which eye movements could not reliably be tracked were excluded from the analyses. This resulted in the exclusion of 9.4% of all trials (3.6% for native English speakers, 5.8% for non-native English speakers).

### Data Processing

All the participants achieved above 90% of gaze samples (calculated by dividing the number of eye-tracking samples that were correctly identified by the number of attempts) with a mean percentage of 95.26%, indicating that they were consistently looking at the visual stimuli during the experiment. For each participant, we exported the raw eye gaze data (timestamp and gaze tracking data) using Tobii Pro Studio software.

Our analyses focused on language-mediated visual attention. Raw eye-tracking data were aggregated into proportions of fixations first by-subjects and then by-items for nine critical time windows, on a trial-by-trial basis, in the linguistic stimuli (see an example in Table 2). We conducted statistical analyses during the time window spanning from the onset of a critical time window in the linguistic stimulus (e.g., onion) +200 ms until its offset +200 ms. We selected the time window of a critical word +200 ms since previous studies have demonstrated that the competition effects of related objects were observed around 200–300 ms after the onset of the target word (e.g., Huettig and Altmann, 2005; Yee and Sedivy, 2006).

**TABLE 2:** Results of linear mixed models.

Time window	Example stimulus	Mean duration (ms)	Native speakers						Non-native speakers					
			By-Subjects			By-Items			By-Subjects			By-Items		
			β	χ2	p	β	χ2	p	β	χ*2*	*p*	β	χ2	*p*
1	*The rabbit is*	164	–0.00	0.00	0.979	–0.00	0.01	0.922	0.00	0.08	0.771	0.00	0.02	0.885
2	*weighing/chopping*	525	0.02	2.12	0.145	0.01	0.73	0.393	0.01	0.34	0.560	0.01	0.27	0.604
3	*the*	105	0.07	18.77	< 0.001	0.05	7.06	0.008	0.02	3.44	0.063	0.02	1.60	0.205
4	*onion*,	414	0.09	46.23	< 0.001	0.08	18.75	< 0.001	0.05	18.91	< 0.001	0.05	6.80	0.009
5	*not*	243	0.11	43.75	< 0.001	0.08	13.91	< 0.001	0.09	42.73	< 0.001	0.08	13.42	< 0.001
6	*smelling*	556	0.08	20.63	< 0.001	0.07	12.46	< 0.001	0.14	55.90	< 0.001	0.10	20.63	< 0.001
7	*the*	100	0.06	11.81	< 0.001	0.05	9.07	0.003	0.08	30.90	< 0.001	0.07	11.05	< 0.001
8	*onion.*	373	0.06	9.51	0.002	0.05	8.77	0.003	0.09	28.14	< 0.001	0.08	14.87	< 0.001
9	+ 500 ms at the offset of linguistic stimuli	500	0.04	4.52	0.033	0.03	4.08	0.044	0.09	29.32	< 0.001	0.08	13.21	< 0.001

*Nine time windows in the linguistic stimuli were used for statistical analyses. 200 ms was added after both the onset and offset of each time window. In the 1st time window, Object-state was included as a fixed effect, participants as random effects in the by-subject model, and items as random effects in the by-item model. In the 2nd to 9th time windows, Degree of Change, Object-state, their interaction were included as fixed effects, participants as random effects in by-subject models, and items as random effects in by-item models.*

We transformed the proportion of fixations for each time window using the arcsine square root transformation to account for the bounded nature of binomial responses (e.g., Williams et al., 2019). We then fit linear mixed models for data of each time window using the *lmer* function in the *lme4* package (Bates et al., 2015) of R (R Core Team, 2020). We assigned sum-coded contrasts to Degree of Change (minimal change = −1; substantial change = 1) and Object-state (intact-state = −1; changed-state = 1).

In the 1st time window, we included Object-state as a fixed effect, participants as random effects in the by-subject model, and items as random effects in the by-item model. In the 2nd–9th time windows, we included Degree of Change, Object-state, their interaction as fixed effects, participants as random effects in by-subject models, and items as random effects in by-item models.^1^ See example models below:

By-subject<-lmer(Trans_Prop∼Degree-of-Change*Object -state + (1| Subject), data = T2)
By-item<-lmer(Trans_Prop ∼ Degree-of-Change*Object-state + (1| Item), data = T2)

We did not fit maximal models due to convergence problems across more complex models in later time windows. To assess the goodness of fit, we compared the models using the χ^2^-distributed likelihood ratio and its associated *p-*value. The model with a smaller Akaike Information Criterion (AIC) and the Bayesian Information Criterion (BIC) was considered as a better fit (Baayen et al., 2008). Only effects that were significant in both by-subject and by-item analyses were accepted as significant. Significant interaction effects between fixed effects were followed by pairwise comparisons with “tukey” adjustment for multiple comparisons using *emmeans* package (Lenth, 2018).

## Results

**FIGURE 2:**
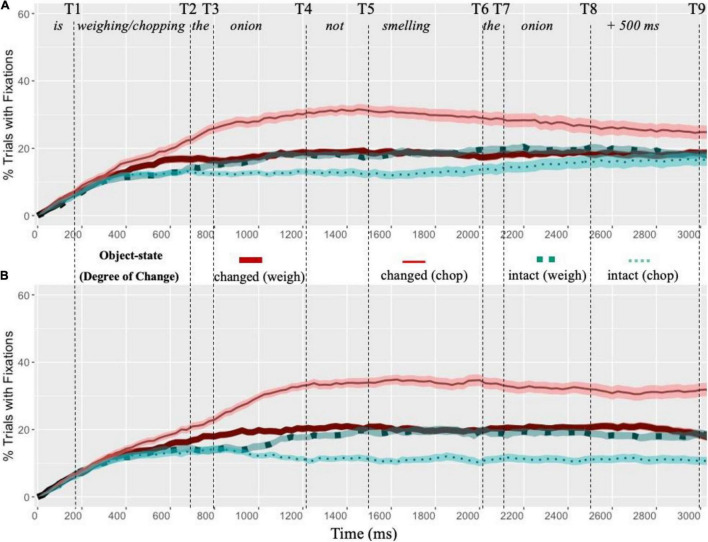
Percentage of trials with fixations launched on the interest areas (AOIs) across sentential conditions. **(A)** Fixations of native speakers. **(B)** Fixations of non-native speakers. The *x*-axis shows the elapsed time increments from the onset of linguistic stimuli (e.g., “*The rabbit is weighing/chopping the onion, not smelling the onion*”). The *y*-axis shows percentage of trials with at least one fixation on the AOIs. Standard errors above and below the mean were shown as shaded areas. The dashed lines indicate the offset of critical time windows.

Figure 2 presents the percentage of trials with fixations on the competing object-states as language unfolded. Table 2 presents results of statistical analyses during 9 critical time windows.

### Native Speakers of English

During the 1st time window, native speakers of English showed no differences in their proportions of fixations to the intact state and the changed state. During the 2nd time window when the critical verbs (e.g., *weighing/chopping*) were mentioned, there was not yet an interaction effect between Object-state and Degree of Change. The first significant interaction effect was found in the 3rd (“*the*”) and in all following time windows. Pairwise comparisons suggested that after hearing a substantial change (e.g., “*chopping*”) (SE condition) than a minimal change (e.g., “*weighing*”) (ME condition) participants initiated higher proportions of fixations toward the changed-state (e.g., a chopped onion) from the 3rd time window to the 9th time window [By-Subjects: *p* < 0.001 (3rd–7th), *p* = 0.006 (8th), *p* = 0.046 (9th); By-Items: *p* = 0.001 (3rd), *p* < 0.001 (4th, 5th), *p* = 0.002 (6th), *p* = 0.036 (7th), *p* = 0.003 (8th), *p* = 0.039 (9th)]. No such differences were found in the proportions of fixations toward the intact state. Thus, despite directing their visual attention toward the changed state of the target object, native English speakers did not show anticipatory eye movements when they just heard the critical verb (e.g., *chop vs. weigh*). The earliest time window revealing such differences in visual attention was during the determiner (“*the”)* right after the critical verb.

### Non-native Speakers of English

Similar to native speakers, non-native speakers did not show any differences in eye movements between the intact-state and the changed-state in the 1st time window. There was no interaction effect between Object-state and Degree of Change in the 2nd time window either. However, unlike native speakers, non-native speakers showed no interaction effect between Object-state and Degree of Change in the 3rd time window (“*the*”). The first interaction effect was found during the 4th (e.g., “*onion*”) and in all following time windows. Pairwise comparisons suggested that there were higher proportions of fixations to the changed-state after a substantial change (e.g., “*chopping*”) (SE condition) than a minimal change (e.g., “*weighing*”) (ME condition) was described [By-Subjects: *p* < 0.001 (3rd–9th); By-Items: *p* = 0.016 (3rd), *p* = 0.007 (4th), *p* < 0.001 (5th, 6th), *p* = 0.003 (7th, 8th), *p* = 0.002 (9th)]. By contrast, no such differences in visual attention were found on the intact state. Thus, compared with native speakers non-native speakers showed a short delay in linguistically mediated visual attention toward the implied end-state of the target object.

## Discussion

The present study investigated how language comprehenders keep track of implied object-states during real-time language processing. We revealed that both native and non-native speakers of English speakers activated and updated object-states in real-time language comprehension. Both groups did not show any anticipatory eye movements at the verb region (e.g., “*chopping/weighing*”), but directed visual attention to the end-state of the target object when they heard the object name (e.g., “*onion*”). In principle, participants could have moved their eye movements toward the expected end-state of the target object as soon as the critical verb was heard. One possibility for the lack of anticipatory eye movement during the verb region is that the competing object-states of the target object on the visual display cannot be integrated with the linguistic context until the specific cue (e.g., “*the onion*”) is provided. Anticipatory eye movements on the visual scene may reflect the integration of linguistic, visual, and world knowledge (Smith and Levy, 2013; Nieuwland et al., 2020). Participants may not be motivated to look to one or the other depiction of the target object as a specific token of *the target object* until the object name was directly referred to.

It is also possible that the intact-state and the changed-state may be two discrete episodic tokens of the target object on the continuum of trajectories in event representations. Preferential looks to object-states may reflect a featural overlap between the visual depiction and mental representations of the target object. In this process, participants may have to go through a multi-step process, in which they first activate the initial state of the target object that affords for the action before activating its intermediate states and the end-state. Only after the verb is specified, they are then able to update mental models of the change-of-state event and thus direct their visual attention to the end-state. A similar hypothesis, known as the two-step hypothesis, was proposed to understand “negation” (e.g., “*The door is not closed*”). According to the two-step hypothesis, we have to first activate the state of affairs before the negation (a closed door) and then the negated state (an open door) (e.g., Kaup and Zwaan, 2003; Kaup et al., 2006; Lüdtke et al., 2008). We postulate that something analogous might be going on when we keep track of object-states in language processing.

However, despite these similarities, non-native speakers showed differences from native speakers in the time course of activating the implied object-state. Only native speakers but not non-native speakers of English directed their visual attention to the end-state of the target object during the determiner region (“*the*”). Our results thus support the alternative hypothesis that native and non-native speakers showed differences in activating mental representations of objects during real-time language processing.

This short delay of visual attention toward the implied object-state among non-native speakers could be accounted for by the *Reduced Ability to Generate Expectations* (*RAGE*) account (Grüter et al., 2017). According to the RAGE account, even advanced non-native speakers were less likely to rely on predictive mechanisms at the discourse level to the same extent as native speakers (see also Kaan et al., 2010; Kaan, 2014). Therefore, non-native speakers may not be able to show the pre-nominal prediction effect (Fleur et al., 2020; Bañón and Martin, 2021), thus they have to launch anticipatory eye movements toward the implied object-state when the object was explicitly mentioned.

However, we could not exclude the possibility that morphosyntactic differences between the L1 (Cantonese) and the L2 (English) of non-native speakers might lead to this delay. Cantonese and English are typologically divergent and genetically unrelated languages (Matthews and Yip, 2011). The change-of-state events were coded differently in Cantonese and English. For example, in English, the verb “*break*” indicates both the action and the consequences, but in Cantonese, they have to be specified separately using the serial verb construction (Francis and Matthews, 2006). Another difference between Cantonese and English is that there is no determiner such as “the” in Cantonese, but classifiers are used before nouns (Chow and Chen, 2020). Thus, further studies may examine whether these morphosyntax differences in L1 and L2 will slow down non-native speakers’ activation of mental representations of event knowledge in real-time language comprehension.

In conclusion, our study demonstrated that both native and non-native speakers of English kept track of object-state representations in real-time language comprehension. They all directed their visual attention toward the end-state of the target object when the object name was directly referred to, but no anticipatory eye movements were found during the verb region. Nonetheless, native speakers but not non-native speakers showed anticipatory eye movements during the determiner (“*the*”). Such similarities between native and non-native speakers in real-time language processing indicate that non-native speakers do not differ significantly from native speakers in how predictive mechanisms are employed for event representations in real-time processing. Our study provides empirical evidence that native-like processing of event knowledge is possible among non-native speakers during real-time language comprehension but a short delay can be observed.

## Data Availability Statement

The datasets presented in this study can be found in online repositories. The names of the repository/repositories and accession number(s) can be found below: https://osf.io/9vb7s/.

## Ethics Statement

The studies involving human participants were reviewed and approved by the Ethics Committee of the Chinese University of Hong Kong. The patients/participants provided their written informed consent to participate in this study.

## Author Contributions

XK: conceptualization, design of sentence stimuli, design of picture stimuli, analysis and interpretation of data, and draft and revision of the manuscript. HG: design of picture stimuli, data acquisition, and draft and revision of the manuscript. Both authors contributed to the article and approved the submitted version.

## Conflict of Interest

The authors declare that the research was conducted in the absence of any commercial or financial relationships that could be construed as a potential conflict of interest.

## Publisher’s Note

All claims expressed in this article are solely those of the authors and do not necessarily represent those of their affiliated organizations, or those of the publisher, the editors and the reviewers. Any product that may be evaluated in this article, or claim that may be made by its manufacturer, is not guaranteed or endorsed by the publisher.

## Footnotes

1 We also analyzed the data using linear mixed models by including Object-state, Degree of Change, and their interactions as fixed effects, and both participants and items as random effects by following Kang et al. (2020). During the 3rd time window, there was a significant interaction among native speakers (χ^2^ = 5.70, *p* = 0.017), but not among non-native speakers (χ^2^ = 0.037, *p* = 0.848).
